# The Importance of Continuous Monitoring in Identifying Bradycardia during Propranolol Treatment for Infantile Hemangiomas

**DOI:** 10.31662/jmaj.2025-0213

**Published:** 2025-11-28

**Authors:** Yasuhiko Maki, Hiroyuki Iijima, Kazue Yoshida, Akira Ishiguro

**Affiliations:** 1 Department of General Pediatrics & Interdisciplinary Medicine, National Center for Child Health and Development, Tokyo, Japan; 2 Center for Postgraduate Education and Training, National Center for Child Health and Development, Tokyo, Japan; 3 Division of Dermatology, National Center for Child Health and Development, Tokyo, Japan; 4 Department of Pediatrics, NHO Disaster Medical Center, Tokyo, Japan

**Keywords:** bradycardia, infantile hemangioma, propranolol, monitoring

## Abstract

**Introduction:**

To clarify whether continuous-monitoring can detect bradycardia during propranolol treatment for infantile hemangioma (IH) and explore management practices for patients with bradycardia.

**Methods:**

This retrospective study with historic controls was conducted on children with IH aged 0-1 year admitted for propranolol treatment at the National Center for Child Health and Development between October 2016 and July 2023. Patients were divided into two groups based on the monitoring method, namely, the spot-measurement group (October 2016 to August 2018) and the continuous-monitoring group (September 2018 to July 2023). Bradycardia was defined as a heart rate of <90/min lasting for 20 minutes. Patient data included clinical characteristics, propranolol dosage, and adverse effects (bradycardia, hypotension, and hypoglycemia). Statistical analyses were performed using Fisher’s exact and Mann-Whitney *U* tests.

**Results:**

During the study period, 106 patients were admitted for propranolol therapy; 49 were in the spot-measurement group and 57 in the continuous-monitoring group. The frequency of bradycardia was significantly higher in the continuous-monitoring group than in the spot-measurement group (21% vs. 2%, p = 0.003). In the continuous-monitoring group, 2 of 12 patients with bradycardia were symptomatic. All patients experienced prompt resolution of symptoms with the reduction of propranolol dosage and had favorable outcomes for IH.

**Conclusions:**

Continuous-monitoring can detect bradycardia more effectively during propranolol treatment for IH than spot-measurement, and reducing the dosage of propranolol can lead to favorable outcomes for IH while minimizing the risk of bradycardia.

## Introduction

Infantile hemangioma (IH) is the most common tumor, occurring in 1%-10% of infants. It is characterized by the abnormal proliferation of endothelial cells and atypical vascular structures ^(1), (2), (3)^. IHs are often absent or small at birth and grow markedly between 1 and 2 months of age, usually converging in growth by 9-12 months. Although IH usually shows a self-limited course of resolution during school age, it sometimes causes bleeding, ulceration, functional impairment, and permanent deformity ^(1), (4)^.

Oral propranolol, a non-selective β-blocker, has the effect of resolving IH and recently replaced systemic corticosteroids as the first-line treatment for problematic IH ^(5), (6), (7), (8)^. Propranolol is usually introduced at mean age of 86-108 days ^(5), (9), (10)^. Propranolol, like other β-blockers, potentially has the effect of heart rate reduction. Most previous studies have reported that bradycardia as an adverse effect rarely occurs with propranolol treatment of IH ^(11), (12), (13)^, although some cases of severe complications due to bradycardia have been reported ^(14), (15)^. Previous studies evaluated propranolol-induced bradycardia using only spot or within a few hours’ measurements of pulse after propranolol initiation.

This study aimed to clarify whether continuous-monitoring can detect bradycardia during propranolol treatment for IH compared to spot-measurement. We also investigated the characteristics of the patients with bradycardia and explored management practices.

## Materials and Methods

### Study design and participants

This retrospective study with historic controls included children with IH admitted to the National Center for Child Health and Development (NCCHD), a tertiary medical institution, from October 2016 to July 2023. At NCCHD, all children with IH requiring propranolol treatment are admitted. Eligible children were aged 0-1 year and were hospitalized for propranolol treatment initiation. The indication for hemangioma is determined by experts in diagnosing, treating, and managing IH (pediatricians and pediatric dermatologists). The patients underwent a pediatric cardiology examination, electrocardiography, and echocardiography before initiating propranolol treatment. Exclusion criteria included children with bronchial asthma, bronchospasm, hypoglycemia, bradycardia, arrhythmia, heart failure, angina, hypotension, pheochromocytoma, severe peripheral circulatory disorders (such as Raynaud’s syndrome), had received prohibited medications (systemic steroids, imiquimod, vincristine, interferon-α, and β-blockers), had other previous procedures for IH, or those with a corrected age of <5 weeks were excluded from this study. Bradycardia was defined as <90/min and hypotension was defined as systemic blood pressure <60 mmHg, according to the Pediatric Advanced Life Support guidelines ^(16)^. This study was performed in line with the principles of the Declaration of Helsinki. Informed consent was obtained from all the patient’s parents with opt-out possibility. Approval was granted by the Ethics Committee of the NCCHD (Date June 11, 2023, No. 2023-146).

### Propranolol initiation protocol

Following a Japanese multicenter, open-label phase III study ^(10)^, propranolol dosing was initiated at 1.0 mg/kg/day in two divided doses twice daily and increased in 1.0 mg/kg/day increments every second day up to target doses during the titration period. The pediatrician and pediatric dermatologist determined the target dose of propranolol to be 3.0 mg/kg/day or 2.0 mg/kg/day. There are reports that 2.0 mg/kg/day is effective in Japanese people, but if there were a high possibility of anatomical functional abnormalities due to the number and size of lesions, the target dose was set at 3.0 mg/kg/day ^(9)^. If adverse effects occurred during treatment, the dose of propranolol was reduced or discontinued to a level where no adverse effects were observed. To avoid the risk of hypoglycemia, propranolol was administered after breastfeeding or meals, with a minimum interval of 9 hours between the morning and evening intakes. Blood glucose measurements during hospitalization were taken before, 1 hour after, and 2 hours after propranolol administration, at the start, and when the dose was increased. Blood pressure was spot-checked three times a day within the windows of 06:00-07:00, 10:00-11:00, and 18:00-19:00, as continuous-monitoring by an arterial line was invasive and difficult to manage. During dose escalation, additional measurements of blood pressure were performed before and at 1 and 2 hours after propranolol administration, corresponding to the expected peak plasma concentration of propranolol. From October 2016 to August 2018, the heart rate was measured using the same method as the blood pressure, and the duration of abnormal heart rate was not considered in the definition of bradycardia. From September 2018 to July 2023, the heart rate was continuously monitored for 24 hours throughout the hospitalization using the Central Monitor CNS-2101 (Nihon Kohden, Japan), and bradycardia was defined as <90/min lasting for 20 minutes. We defined the period from October 2016 to August 2018 as the spot-measurement period and the period from September 2018 to July 2023 as the continuous-monitoring period.

### Outcome measures

We collected the following from electronic medical records: medical history, corrected age at admission, sex, weeks of gestation, birth weight, adverse effects (bradycardia, hypotension, and hypoglycemia), target dose of propranolol, and final dosage of propranolol. In the continuous-monitoring group, bradycardia was judged to be present when bradycardia lasted longer than 20 minutes.

#### Statistical analysis

We compared the frequency of bradycardia and patient characteristics between patients during the spot-measurement period and the continuous-monitoring period. In addition, we investigated the clinical course of patients who experienced bradycardia during the continuous-monitoring period. Categorical variables are presented as numbers or percentages, and continuous variables are presented as median values with interquartile ranges (IQRs). Categorical and continuous data were compared using Fisher’s exact and Mann-Whitney *U* tests. Two-sided p < 0.05 were considered statistically significant. All data were analyzed using SPSS software version 29 (IBM Corporation, Armonk, NY, USA).

## Results

During the study period, a total of 106 patients were admitted for propranolol therapy; 49 were in the spot-measurement group and 57 were in the continuous-monitoring group. Female patients represented 75%, and 54 patients (51%) had a target dose of propranolol of 3.0 mg/kg/day. The clinical characteristics and adverse effects of the patients are summarized in Table 1. On admission, patients in the continuous-monitoring group were younger (median age 11 weeks [IQR 9-14] vs. 15 weeks [IQR 10-17], p = 0.03) than those in the spot-measurement group. No patients had echocardiographic or electrocardiographic results that precluded propranolol induction. The frequency of bradycardia was significantly higher in the continuous-monitoring group than in the spot-measurement group (12 of 57 patients [21%] vs. 1 of 49 patients [2%], p = 0.003). Hypotension occurred in one patient (2%) in the continuous-monitoring group and none in the spot-measurement group, and hypoglycemia did not occur in either group.

**Table 1.** Clinical Characteristics and Adverse Effects of Patients Treated with Propranolol.

Characteristics	Continuous measurement		Spot measurement		p Value
	n or median	(% or IQR)	n or median	(% or IQR)	
	N = 57		N = 49		
Female	45	(79)	34	(69)	0.27
Gestational age (weeks)	39	(38-40)	39	(38-40)	0.94
Birth weight (kg)	2.94	(2.69-3.22)	3.02	(2.69-3.26)	0.48
Age (weeks) at admission	11	(9-14)	15	(10-17)	0.03
Target dose 3.0 mg/kg	31	(54)	23	(47)	0.56
Laser therapy	14	(25)	13	(27)	0.83
Bradycardia	12	(21)	1	(2)	0.003
Hypotension	1	(2)	0	(0)	>0.99
Hypoglycemia	0	(0)	0	(0)

IQR: interquartile range.

Among the 57 patients in the continuous-monitoring group, age at admission (median 14 weeks [IQR 11-23] vs. 11 weeks [IQR 7-13], p = 0.01) was higher in patients with bradycardia (Table 2). There were no significant differences in sex, weeks of gestation, birth weight, and target dose of propranolol. The clinical characteristics of the 12 patients with bradycardia in the continuous-monitoring group are summarized in Table 3. All patients had a favorable outcome for IH. The final dosage of propranolol for patients with bradycardia was 0.06-2.0 mg/kg/day, and no patients discontinued treatment during the induction hospitalization. We described two patients (cases 5, and 10 in Table 3) with an interesting clinical course out of 12 with bradycardia.

**Table 2.** Characteristics of Patients in the Continuous Measurement Group.

Characteristics	Bradycardia		No bradycardia		p Value
	n or median	(% or IQR)	n or median	(% or IQR)	
	N = 12		N = 45		
Female	10	(83)	35	(78)	>0.99
Gestational age (weeks)	39	(38-40)	39	(38-40)	0.84
Birth weight (kg)	3.24	(2.80-3.33)	2.81	(2.60-3.11)	0.09
Age (weeks) at admission	14	(11-23)	11	(7-13)	0.01
Target dose 3.0 mg/kg	8	(67)	23	(51)	0.52

IQR: interquartile range.

**Table 3.** The Clinical Characteristics of Twelve Patients with Bradycardia.

Case	Sex	Birth weight (g)	Gestational age	Propranolol initiation age	Location (size [mm])	Propranolol dose (mg/kg/day)			Other adverse effects	Outcome of infantile hemangioma
						Target	Bradycardia	Maintenance		
1	F	3618	40w0d	4m2d	Nasal bridge (10 × 4)	2.0	1.0	0.5	None	Favorable
2	F	3316	38w0d	4m23d	Head (50 × 50)	3.0	2.0	2.0	None	Favorable
3	F	3206	39w2d	2m8d	Labia majora (30 × 15)	3.0	3.0	2.0	None	Favorable
4	F	3270	38w3d	2m10d	Cheek (11 × 13), Abdomen (4 × 4)	2.0	1.0	0.06	None	Favorable
5	M	2738	40w5d	5m14d	Forehead (4 × 4), Arm (35 × 25), Chest (1 × 1), Buttock (25 × 8)	3.0	2.0	0.5	Apnea	Favorable
6	F	3292	40w2d	2m21d	Nasal tip (8 × 10)	3.0	1.0	0.3	None	Favorable
7	F	1600	35w6d	2m27d	Head (24 × 21)	2.0	1.0	0.85	None	Favorable
8	F	3072	38w3d	2m13d	Head (15 × 15)	2.0	1.0	0.4	None	Favorable
9	M	2196	37w6d	2m28d	Arm (80 × 45)	3.0	1.0	1.0	None	Favorable
10	F	2968	38w5d	3m20d	Upper lip (12 × 8)	3.0	3.0	2.0	Lethargy	Favorable
11	F	3460	39w4d	8m27d	Orbit (29 × 30)	3.0	1.0	0.26	None	Favorable
12	F	3338	40w6d	5m20d	Arm (no data)	3.0	2.0	1.0	None	Favorable

d: day; F: female; m: month; M: male; w: week.

**Figure 1.**
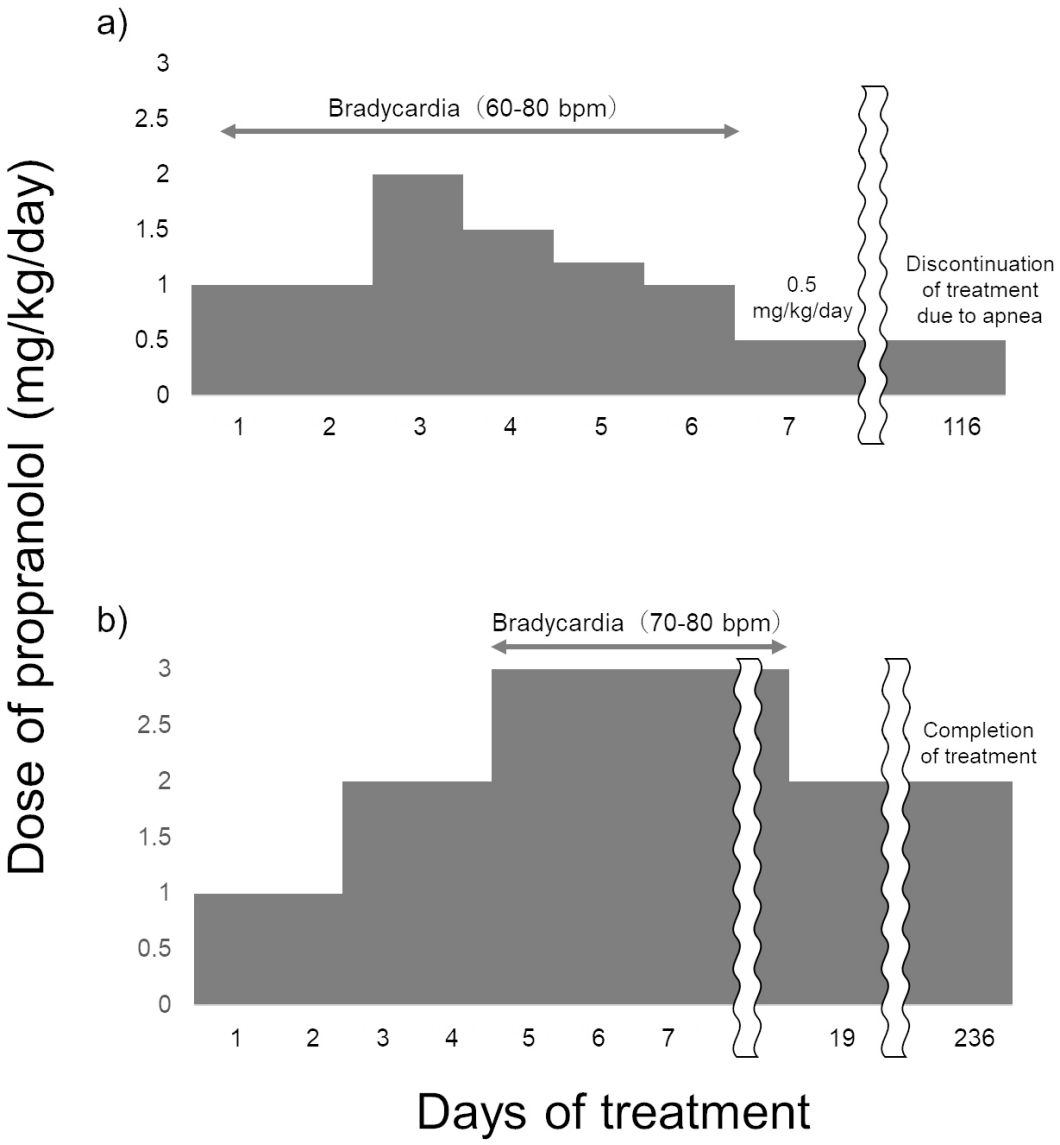
Clinical course of treatment of infantile hemangioma with propranolol (a: case 5, b: case 10).

Case 5: A 5-month-old boy with no significant medical history presented with multiple superficial IHs on the face (4 × 4 mm), forearms (35 × 25 mm), chest (1 × 1 mm), and buttocks (25 × 8 mm). From the third day of admission, when propranolol was increased to 2.0 mg/kg/day, he had bradycardia with a heart rate <70/min. Finally, he was discharged without bradycardia at a maintenance propranolol dose of 0.5 mg/kg/day. At 10 months of age, he was admitted for evaluation of recurrent apnea. On monitoring, he was found to have a heart rate <50/min, oxygen saturation as measured by pulse oximetry decreasing to 80%, and apnea during sleep. Propranolol was discontinued, and the apnea subsequently resolved without bradycardia or decreased SpO_2_. Because the apnea resolved completely only with the discontinuation of propranolol and did not recur thereafter, no additional evaluation, including airway imaging, was performed. At this point, the IHs resolved sufficiently, and treatment was discontinued (Figure 1a).

Case 10: A 3-month-old girl with no significant medical history presented with a superficial IH on the upper lip (12 × 8 mm). On the fifth day of admission, the propranolol dose was escalated to 3.0 mg/kg/day. Following dose escalation, she became less active and developed bradycardia during sleep. The blood glucose level was above 90 mg/dL when decreased activity was observed. On the 19th day, the dose of propranolol was reduced to 2.0 mg/kg/day, after which her arousal level returned to normal. Subsequently, her treatment progressed favorably with no adverse effects (Figure 1b).

## Discussion

In this study, the frequency of bradycardia in the continuous-monitoring group was 21%, significantly higher than the 2% in the spot-measurement group. The age of patients with bradycardia was higher than that of those without bradycardia. Some patients with bradycardia were symptomatic, presenting with apnea or lethargy. Although this study cannot establish a causal relationship between bradycardia and these symptoms, the symptoms appeared concurrently with bradycardia and resolved following dose reduction of propranolol. Therefore, we considered these symptoms to be associated with bradycardia.

Previous studies of IH have shown that the frequency of bradycardia caused by propranolol is <1.0%, including in asymptomatic patients ^(10), (17)^. These studies used spot-measurements to determine bradycardia, and the frequencies of bradycardia were nearly identical to the spot-measurement group in our study. From the perspective of medical costs, some reports recommend initiating treatment with spot-measurements of vital signs in an outpatient setting ^(18), (19)^. However, our findings suggest that initiating treatment under continuous-monitoring is preferable for detecting bradycardia and for the appropriate management of adverse side effects. While cost-effectiveness must also be considered, patient safety is the highest priority. Further research is needed to establish an initiation protocol that optimizes the balance between safety and resource utilization.

In our study, patients in the continuous-monitoring group were younger than those in the spot monitoring group. This trend may reflect early therapeutic intervention for IH at our institution. In contrast, the younger age of the patients in the continuous-monitoring group should not affect the results of our study as patients with bradycardia were older than those without bradycardia. Although the Japanese guidelines identify infants aged <90 days or weighing <5 kg as being at higher risk of adverse events during propranolol treatment ^(20)^, our study found that bradycardia occurred more frequently in older infants, and there was no significant difference in body weight between those with and without bradycardia. This discrepancy may be attributed to differences in the methods and types of adverse events evaluated. The study on which the guidelines are based reported adverse events in 2.1% of patients, with the majority being sleep disturbances (65%) and agitation (11%), and did not include a detailed evaluation of bradycardia or other vital signs ^(21)^. In contrast, our study employed continuous-monitoring, enabling the assessment of vital signs, which may have revealed a different pattern of adverse events.

Infants presenting with bradycardia may manifest nonspecific symptoms, such as feeding difficulties and decreased level of consciousness, with risks of syncope and seizures due to sudden declines in cerebral perfusion ^(22), (23)^. A previous study has described an infant patient with IH who developed bradycardia and severe apnea requiring resuscitation during treatment with propranolol, resolving upon discontinuation of propranolol ^(15)^. In our study, 2 of 12 patients with bradycardia were symptomatic, with one presenting with severe apnea. These symptoms promptly resolved with the reduction or discontinuation of propranolol. Early detection of bradycardia through continuous-monitoring may prevent the progression to severe adverse events such as bradycardia or apnea requiring resuscitation. All 12 patients with bradycardia had favorable treatment courses for IHs even after dose reduction of propranolol. These findings suggest that reducing the dose of propranolol while continuing its administration may be effective in treating IHs with minimal adverse effects when bradycardia occurs.

This study has some limitations. The present study was a single-center design, and all participants were Japanese. Pharmacokinetic studies have shown that propranolol systemic exposure is highly variable depending on the variants of the *CYP2D6* gene involved in drug metabolism ^(24)^, and that interindividual variability in peak plasma concentrations of orally administered propranolol and racial differences in drug sensitivity exist ^(25)^. Therefore, we should be cautious about immediately applying our results to other communities. However, given that no bradycardia was detected by spot-measurement in the Japanese multicenter, open-label phase III study of propranolol ^(10)^, spot-measurements may miss bradycardia, even if Japanese individuals are more susceptible to propranolol.

In conclusion, bradycardia may occur more frequently during propranolol treatment for IH than previously considered and can be detected by continuous-monitoring. Reducing, rather than discontinuing, the dosage of propranolol can help patients achieve favorable outcomes while avoiding bradycardia.

## Article Information

### Acknowledgments

The authors are grateful to the professional native English editor of the Center for Postgraduate Education and Training, NCCHD, for English language editing.

### Author Contributions

Yasuhiko Maki: Conceptualization, Methodology, Formal analysis, Investigation, Data curation, Writing - Original Draft, Visualization.

Hiroyuki Iijima: Conceptualization, Methodology, Formal analysis, Investigation, Data curation, Writing - Review & Editing, Visualization, Project administration.

Kazue Yoshida: Conceptualization, Investigation, Writing - Review & Editing

Akira Ishiguro: Conceptualization, Methodology, Writing - Review & Editing, Visualization, Supervision.

### Conflicts of Interest

Kazue Yoshida received funding from Maruho Company, Ltd. and also received payment or honoraria for lectures and presentations from Maruho Company, Ltd. This company was not involved in (1) the study design; (2) the collection, analysis, and interpretation of data; (3) the writing of the report; or (4) the decision to submit the paper for publication. Other authors declare no conflicts of interest.

### IRB Approval Code and Name of the Institution

Approval was granted by the Ethics Committee of the National Center for Child Health and Development (NCCHD, Date 6/11/2023, No2023-146).
